# A survey: which features are required for dynamic visual simultaneous localization and mapping?

**DOI:** 10.1186/s42492-021-00086-w

**Published:** 2021-07-16

**Authors:** Zewen Xu, Zheng Rong, Yihong Wu

**Affiliations:** 1 School of Artificial Intelligence, University of Chinese Academy of Sciences, Beijing, 100049 China; 2 National Laboratory of Pattern Recognition, Institute of Automation, Chinese Academy of Sciences, Beijing, 100190 China

**Keywords:** Dynamic simultaneous localization and mapping, Multiple objects tracking, Data association, Object simultaneous localization and mapping, Feature choices

## Abstract

In recent years, simultaneous localization and mapping in dynamic environments (dynamic SLAM) has attracted significant attention from both academia and industry. Some pioneering work on this technique has expanded the potential of robotic applications. Compared to standard SLAM under the static world assumption, dynamic SLAM divides features into static and dynamic categories and leverages each type of feature properly. Therefore, dynamic SLAM can provide more robust localization for intelligent robots that operate in complex dynamic environments. Additionally, to meet the demands of some high-level tasks, dynamic SLAM can be integrated with multiple object tracking. This article presents a survey on dynamic SLAM from the perspective of feature choices. A discussion of the advantages and disadvantages of different visual features is provided in this article.

## Introduction

For intelligent robots to work with humans, robots must be able to determine their own locations. Simultaneous localization and mapping (SLAM) is a common method for addressing this problem. SLAM is considered as an important technique for intelligent robot self-localization, particularly in areas lacking global position information, such as tunnels and indoor scenes. The main problems associated with vision-based SLAM (V-SLAM) are the extraction and matching of a series of visual features from image sequences with temporal relationships and how to utilize these features to estimate the camera pose and construct a consistent three-dimensional (3D) structure of an unknown scene simultaneously. To tackle these problems, V-SLAM systems generally contain a set of common blocks, including feature tracking, map building, and loop closure detection for error drift correction. The implementation details of these modules vary according to many factors, including the employed visual sensor types, utilization of features, and optimization methods.

Many types of cameras are used for V-SLAM, including monocular cameras, stereo cameras, RGB-depth (RGB-D) cameras, and event-based cameras. SLAM using only a monocular camera cannot estimate global scale directly and must rely on additional sensors such as inertial measurement units (IMUs) or additional priors such as shape priors to overcome scale ambiguity. However, a monocular camera is the most appropriate choice for weight-constrained micro-aerial vehicles based on its light weight, low power requirements, and attractive price. In comparison, RGB-D SLAM can obtain depth information and estimate global scale directly, but it is extremely sensitive to light, which limits its application in most outdoor scenes. Stereo SLAM can estimate depth and global scale directly based on the length of the baseline between left and right cameras. The accuracy of depth estimation in stereo camera models relies heavily on the length of the baseline, which limits their application in portable mobile devices. Unlike the three conventional camera types mentioned above, event-based cameras are biologically inspired. Events are time-stamped changes in the brightness of independent pixels. Event-based cameras can directly capture events asynchronously, leading to lower latency and a higher dynamic range than conventional cameras. Therefore, event-based cameras can be used to tackle difficult tasks such as rapid and dynamic obstacle avoidance [1].

According to the amount of feature information used for matching, visual features can be divided into two levels: low-level features such as pixel patches, points, or lines, and high-level features such as semantically labeled objects [2]. Different features describe scenes from different perspectives. Low-level features focus on local details such as textures or the geometric primitives of objects and scenes. High-level features integrate details into semantic labels that more closely match the human understanding of the world. This article reviews recent approaches to SLAM in dynamic environments to explore the advantages and disadvantages of different levels of features.

From the perspective of optimization, SLAM can be divided into two classes: filter-based SLAM and frame-based SLAM. The former marginalizes past poses and summarizes the information gained over time using a probability distribution. In contrast, the latter selects only a small number of past frames and applies bundle adjustment (BA) to those frames [3]. Although many frame-based SLAM methods [4–7] have demonstrated that the BA method is more efficient for V-SLAM, filtering methods are still worth studying for dynamic SLAM based on their natural advantages in terms of handling statistical information, which is important for depth estimation [8], sensor fusion [9], dynamic feature determination [10], and robust map management [11, 12].

The V-SLAM problem can be addressed elegantly in static or approximately static textured scenes. In such cases, there are sufficient background features for ego estimation. However, in more complex real-world environments such as crowded corridors or malls, the classical SLAM pipeline yields poor results because it cannot handle dynamic features properly [13]. There are two methods for solving this problem. The first is culling dynamic features/correspondences as outliers, which is known as the robust SLAM problem (Robust sections). The second method is integrating SLAM and multiple object tracking (MOT), which is known as the SLAMMOT problem (SLAMMOT sections). Intuitively, leveraging dynamic features to estimate the camera pose, rather than simply culling them, is more robust and meaningful. This is because the SLAM problem is closely related to object detection [14] and MOT [15]. In other words, these methods can benefit from each other. Object detection and MOT can be separately adopted for feature extraction and data association in high-level-feature SLAM. Conversely, SLAM can promote object detection and MOT based on camera poses and object poses to achieve more accurate detection and tracking.

In terms of real-world applications, estimating the states of objects in views is important for robots to make correct decisions and perform interactions with humans. For example, knowing the states of pedestrians and other cars can help driverless cars make more reliable decisions and prevent traffic accidents. Additionally, MOT provides mobile phones with the ability to render moving objects using augmented reality (AR).

This article reviews visual SLAM in dynamic environments from the perspective of using features. Each level of feature is discussed and compared thoroughly in terms of the major components of dynamic SLAM. Additionally, the potential relationships between SLAM and MOT are analyzed. Furthermore, some key points regarding different cameras and optimization methods for dynamic SLAM will be emphasized. The strengths and weaknesses of each type of feature in dynamic environments are also discussed.

### Existing surveys on dynamic SLAM or its components

Several recent surveys related to dynamic SLAM were reviewed, as shown in Table 1. Xia et al. [18] surveyed semantics-based V-SLAM. Chen et al. [19] discussed the use of deep learning in SLAM. Saputra et al. [16] identified three main problems associated with dynamic SLAM and classified dynamic SLAM into three categories for robust visual SLAM, dynamic object segmentation and 3D tracking, and joint motion segmentation and reconstruction. In a recent survey on feature-based SLAM, Azzam et al. [2] discussed the strengths and weaknesses of various features used in SLAM.

**Table 1:** Recent surveys related to dynamic SLAM

Year	Topic	References
2018	Dynamic SLAM	[16]
2019	Motion segment based on optical flow	[17]
2020	Semantics-based V-SLAM	[18]
2020	Deep learning for SLAM	[19]
2020	Feature-based SLAM	[2]

Dynamic SLAM based on the outstanding works by Saputra et al. [16] and Azzam et al. [2] is also discussed herein. In this paper, features refer to two-dimensional (2D) features and landmarks refer to reconstructed 3D features.

### Article organization

The main problem in dynamic SLAM is handling dynamic data associations. By choosing whether to cull dynamic correspondences or use them to track objects, the dynamic SLAM problem can be considered as a robustness problem or extension of standard SLAM [16]. The remainder of this paper is organized as follows. Low-level-feature-based dynamic SLAM section discusses how to leverage low-level features alone in a dynamic environment. Using high-level features in dynamic SLAM section discusses the functions of high-level features in dynamic SLAM. Finally, the advantages and disadvantages of different levels of features combined with the difficulties of dynamic SLAM are discussed.

Table 2 provides a compact overview of recent robust SLAM systems. There are many difficulties in robust SLAM, including robustly judging dynamic features, handling occlusion, maintaining the long-term consistency of maps, and dealing with few valid point features when dynamic features are culled. The details are discussed in Robust SLAM sections. Comparisons of low-level features and high-level features are provided in the Discussion portion of Robust SLAM section.

**Table 2:** Summary of recent robust SLAM systems

References	System properties			Implementation details		Practical consideration				
	Backbone	CT	Env	MS	HE	P/S	BI	OH	LC	HL
Low-level based SLAM (Robust SLAM section)										
Point-based or pixel-patch-based SLAM										
Yang et al. [20]	ORB-SLAM2 [21]	D	I	RE	–	–	–	–	–	–
Du et al. [22]	ORB-SLAM2	D	I	E + RE	–	√	–	–	√	–
Zhang et al. [23]	–	D	I	OF + DI	–	√	√	–	–	–
Tan et al. [24]	PTAM [6]	M	I	RE	–	–	–	√	–	–
Point-line-based SLAM										
Zhang et al. [25]	–	D	I	3DE	–	√	–	–	√	√
Using high-level feature as semantic priors in low-level feature-based SLAM (Using high-level features as semantic priors for low-level-feature-based SLAM section)										
Point-based SLAM										
Bescos et al. [26]	ORB-SLAM2	M, S, D	I, O	SI + DI	S [27]	–	√	√	√	–
Yu et al. [28]	ORB-SLAM2	D	I	SI + E	S [29]	–	–	–	–	–
Cui and Ma [30]	ORB-SLAM2	D	I	SI + E	S [29]	–	–	–	–	–
Han and Xi [31]	ORB-SLAM2	D	I	SI + OF	S [32]	–	–	–	–	–
Long et al. [33]	ORB-SLAM2	D	I, O	SI + DI	S [32]	–	√	–	–	–
Ai et al. [34]	ORB-SLAM2	S, D	I, O	SI	O [35]	√	–	–	√	–
Xiao et al. [36]	ORB-SLAM2	M	I, O	SI + RE	O [37]	√	–	–	√	–
Brasch et al. [38]	ORB-SLAM [39]	M	O	SI + T	S [40]	√	–	–	√	–
Point-line-based SLAM										
Zhang et al. [41]	–	D	I	SI + DI + E*	O [42]	–	–	–	–	√
Using high-level features in object SLAM (Using high-level features in object SLAM section)										
Yang and Scherer [14]	–	M	I, O	E	O [43]	–	–	–	–	√

System properties: The backbone of the system (Backbone). Camera type (CT): RGB-D (D), monocular (M), stereo (S). Environment (Env): indoor (I), outdoor (O). Implementation details: Method of motion segmentation (MS): reprojection error (RE), epipolar (E), distance between matched and predicted 3D landmarks (3DE), semantic information (SI), depth information (DI), optical flow (OF), triangulation (T). High-level feature extractor (HE): semantic segmentation network (S), object detection network (O). Practical consideration: Use a probabilistic model or dynamic score (wight) to judge dynamic features (P/S). Long-term consistency (LC). Handle low-texture or less static point-feature man-made scenes (HL). *The epipolar constraint is only used on point features

Table 3 provides a compact overview of recent SLAMMOT systems. The main difficulties discussed in this article are missing data handling, relative-scale problem solving for monocular systems, and probabilistic data associations for noisy measurements. The details are discussed in SLAMMOT sections. Comparisons of low-level features and high-level features are provided in the *Discussion* portion of SLAMMOT section.

**Table 3:** Summary of recent SLAMMOT systems

References	System properties					Implementation details				Practical consideration				
	CT	Env	ON	OMT	MK	MMS	HD	HE	OM	HMD	SR	NP	PD	DR
Low-level based SLAM (SLAMMOT section)														
Point-based SLAM														
Wang et al. [44]	S	I	M	R	–	SSC	–	–	J	–	I	√	–	√
Judd et al. [45]	S	I	M	R	–	MMF	–	–	J	–	I	√	–	–
Use high-level features in low-level feature-based SLAM (Using high-level features in point-based SLAM section)														
Point-based SLAM														
Nair et al. [46]	M	O	M	R	C, O	SI	L	S [27]	J	–	√	–	–	–
Huang et al. [47]	S	I, O	M	R	–	SI	L	O [43]	S	√	I	√	√	–
Bescos et al. [48]	S D	O	M	R	–	SI	L	S	J	√	I	√	–	–
Ballester et al. [49]	D	O	M	R	–	SI	L	S [50]	J	√	I	√	–	–
Zhang et al. [51]	M, S, D	I, O	M	R	–	SI	L	S [27]	J	√	-^1^	√	–	–
Using high-level features in object SLAM (Using high-level features in object SLAM section)														
Yang and Scherer [14]	M	I, O	M	R	–	SI	L	O [43]	S	–	–	√	–	–
Qiu et al. [52]	M	I	S^2^	R	C^3^	SI	NN [53]	O [54]	S	–	√	√	–	–
Strecke et al. [55]	D	I	M	R	–	SI	L	S [27]		√	I	√	√	√

System properties: Camera type (CT): RGB-D (D), monocular (M), stereo (S). Environment (Env): indoor (I), outdoor (O). Object number (ON): single (S), multiple (M). Object motion type (OMT): rigid (R), non-rigid (NR), motion knowledge (MK): need knowledge about regarding object motion (O), need knowledge regarding camera motion (C), need no knowledge regarding motion (−). Details: Multi-motion segmentation (MMS): sub-space cluster (SSC), multi-motion fitting (MMF), semantic information (SI). High-level data association for object SLAM (HD) low-level-feature-based method (L), neural-network-based method (NN). High-level feature extractor and for object SLAM (HE): semantic segmentation network (S), object detection network (O). Optimization method (OM): joint optimization (J), separate optimization (S). Practical Consideration: Handle missing data (e.g., due to occlusion, lost tracks, motion blur) (HMD). Solve the relative-scale problem (SR): irrelevant for the type of camera (I). No need for shape priors (NP). Probabilistic data association (PD). Dense reconstruction (DR). 1. Cannot solve the relative-scale problem of monocular cameras; 2. Can implement MOT using multi-region BA; 3. Camera motion information comes from the IMU

## Low-level-feature-based dynamic SLAM

Low-level features mainly include point and line features. Point features are widely employed in SLAM systems based on their outstanding performance for textured scenes. Additionally, classical open-source point-based SLAM systems [21] provide reliable backbones for dynamic SLAM research. Compared to point features, line features contain more geometric structures that can be commonly observed in manmade environments. Although it is feasible to construct a complete SLAM system based on line features alone [56], this yields no significant improvement in performance and often performs worse than point-based SLAM. Recent works [57–59] have demonstrated that leveraging both point and line features can lead to a robust SLAM system based on their complementarity [58]. Therefore, this article mainly focuses on point-based and point-line-based SLAM in dynamic environments.

In static environments, typical methods for point feature matching can be roughly grouped into pixel-based and descriptor-based methods. Pixel-based methods are efficient, but sensitive to illumination changes, which limits their application to long-term data association establishment. Conversely, descriptor-based methods are more robust to illumination changes, so they are widely utilized in the front and back ends of static SLAM systems. To guarantee matching accuracy, typical descriptor-based methods typically leverage motion information and the 3D positions of features to guide matching [21]. However, for dynamic monocular scenarios without additional sensors, it is difficult to predict the movement of landmarks accurately. Therefore, descriptor-based methods cannot establish sufficient dynamic data associations for highly dynamic object tracking. Most existing systems utilize optical flows to address this problem [14]. Based on the development of hardware and deep learning, designing extractors and feature matching systems using data-driven methods has attracted significant attention [60, 61]. In contrast to handcrafted descriptors, learned local descriptors contain more semantic information and perform better on most datasets. Additionally, data-driven methods provide an easy way to combine detection and data association, which frees descriptor-based methods from the burden of motion estimation.

For line feature matching, the combination of a line segment detector [62] and line binary descriptor [63] has been widely applied in many point-line SLAM systems [59]. Additionally, deep learning is also used to design new descriptors for line segments [64]. A higher computational burden is incurred for detecting and matching line segments compared to points. Therefore, tracking lines on moving objects using descriptor-based methods provides low yields. The implementation details of low-level-feature-based dynamic SLAM will be discussed from two perspectives: culling dynamic features (including point and point-line features, Robust SLAM section) or leveraging them (major focus on point features, SLAMMOT section).

### Robust SLAM

To cull dynamic features properly, robust SLAM must distinguish between dynamic and static features, which is known as motion segmentation. This problem can be solved using various approaches. According to the information used for low-level-feature-based dynamic SLAM, existing approaches can be grouped into optical flow methods, geometric methods, and motion-based methods.

Optical flow methods: Optical flows depict the kinematics of features in a 3D space based on the kinematics of their projections in a 2D image space.

For point features, an optical flow is defined by the time derivative of pixel intensity. Such flows are widely applied to track moving objects when a camera is stationary or to estimate the camera pose when an environment is stationary [65]. In a dynamic SLAM system, the camera and objects in an environment may be dynamic. An intuitive approach is to estimate the camera ego motion first and then use the optical flow between the predicted and measured images to detect moving objects. However, this is a type of egg-chicken problem. Zhang et al. [23] proposed a method to overcome this difficulty by estimating the camera ego motion using depth and intensity information in a coarse-to-fine scheme. This motion was then used to compute the scene flow to detect dynamic features.

For line features, Faugeras et al. [66] presented a complete description of the relationship between the 3D and 2D motion of lines. In contrast to the optical flows for point features, straight-line optical flows are represented by the time derivative of the normal to the plane defined by the 2D line and optical center of the camera. This method has been used in multi-motion segmentation [67] and 3D detection [68]. However, thus far, it has not been used in dynamic SLAM.

Geometric methods: These methods set a threshold with geometric constraints for static data associations to detect dynamic features.

**Fig. 1:**
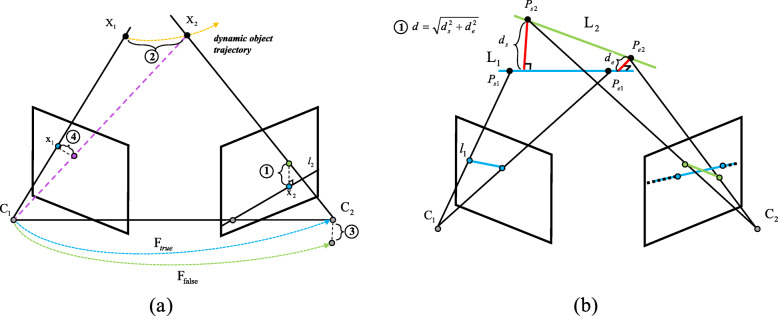
(**a**): The violation of geometric constraints for point features in dynamic environments: (1) the tracked feature lies too far from the epipolar line, (2) back-projected rays from the tracked features do not meet, (3) faulty fundamental matrix estimation occurs when a dynamic feature is included in pose estimation, (4) high distance between re-projected features and observed features [16]; (**b**): The violation of geometric constraints for line features in a dynamic environment: (1) the matched 3D line (green) lies too far from the predicted 3D line (blue)

For point features, constraints can be derived from the equation of epipolar lines [69], back-projected rays (triangulation) [70], camera pose [71] estimation, or re-projection error [72]. First, all features are assumed to be static. Under this assumption, epipolar lines, 3D landmark positions (least square solution), camera poses, or projections can be estimated. Then, the errors between estimates and measurements can be computed and dynamic features can be detected according to a preset threshold. Geometric methods for point features were thoroughly discussed by Saputra and Trigoni [16]. The core of this type of method is illustrated in Fig. 1a.

For line features, Zhang et al. [25] detected dynamic line features using an RGB-D camera. The structures of 3D landmarks corresponding to lines in an image can be reconstructed in the current frame using the depth information captured by an RGB-D camera. They used static point features to obtain the initial camera motion. The poses of lines in a new frame can then be predicted using the initial transformation. Finally, they defined the distance between the matched and predicted 3D lines, as shown in Fig. 1b, and used it to detect dynamic line features. For point-based SLAM, the re-projection error of line features was proposed and has been used for optimization in many point-line-based SLAM systems [67, 73]. However, this geometric information has rarely been used for motion segmentation. To avoid the additional computations introduced into point-based dynamic SLAM by dynamic line feature detection, depth [25] or semantic information [74] is typically used. This type of information can provide more reliable constraints compared to re-projection errors.

Motion-based methods leverage the fact that camera motion can constrain static features. The ego motion information provided by an IMU can easily distinguish static features from dynamic features. This is because only static features conform with IMU information. Additionally, this method can be combined with the two methods discussed above. Kim et al. [75] used an IMU to compensate for the rotation between consecutive images and then computed motion vectors. These dynamic features, which exhibited different tendencies with sensor movement, were filtered according to a threshold. To the best of our knowledge, there are no point-line-based dynamic SLAM method utilizing IMU information. However, such a system could be established by slightly modifying a visual-inertial odometry system based on point and line features [73].

Discussion: Recent systems are listed in Table 2. For point-based robust SLAM, geometric methods can segment features without other priors, but using one geometric threshold alone always results in the problem of motion degeneration. For example, a threshold based on epipolar lines cannot detect dynamic features moving along epipolar lines. In contrast, motion-based methods can easily address motion degeneration. Optical flow methods can be segmented in 2D spaces without other priors. However, they are sensitive to lighting conditions. Therefore, combining two or three of the methods discussed above is a promising alternative approach. When static features are detected, a standard SLAM system (including both direct SLAM [76] and indirect SLAM [77]) can be used to estimate camera ego motion and reconstruct a map of the surrounding scene.

Although point-line-based SLAM has been proven to provide accurate and robust results, few studies have focused on its extension to dynamic environments because of its high computational burdens with relatively low performance improvements. However, line features still have a place in special dynamic environments such as crowded corridors and stairs. Therefore, exploring a more efficient method for extracting and matching line features is important for their application in dynamic scenes.

Additionally, it is not robust to detect dynamic components only across a small number of consecutive frames because the methods discussed above cannot distinguish measurements with noise and slowly moving features based on short-term observations. Du et al. [22] tackled this problem by constructing a probabilistic model and detected dynamic features using conditional random fields (CRFs) with long-term observations, which guaranteed the long-term consistency of maps. Although Zhang et al. [23] used a dynamic score to tackle noisy observations, their system cannot maintain long-term consistency because this score is generated based on one observation with no historical information.

Another problem that must be considered is occlusion. In static cases, the quality of 3D landmarks is defined by the number of observations [7, 21]. Landmarks occluded by a slowly moving object may be culled because of a lack of observations. Therefore, the estimated camera position may drift frequently or be lost [24]. A standard approach to addressing this problem is to detect occlusion. Tan et al. [24] detected occlusion based on the appearance of features and motion information of a camera. They saved those rarely observed landmarks to combat occlusion and improve the robustness of the system.

However, in highly dynamic scenarios, robust SLAM faces the problem of lacking data associations after dynamic features are culled as outliers. In contrast, the useful information contained in dynamic features is leveraged in SLAMMOT [45].

### SLAMMOT

Low-level-feature-based SLAMMOT has two core modules: multi-motion segmentation, and 3D object tracking and reconstruction. The inputs for multi-motion segmentation can be all correspondences or dynamic correspondences only. The outputs are clusters of correspondences with motion labels, which serve as the inputs for 3D object tracking and reconstruction. The outputs of the second module are the trajectories of the camera and dynamic objects, and the structures of the static environment and dynamic objects. Although Zhang et al. [67] proposed a method for performing multi-motion segmentation based on line features, no dynamic SLAM systems have tracked line features. Therefore, a point-based method for solving this problem is discussed.

#### Multi-motion segmentation

The premise of using dynamic features in SLAM system is to classify them according to their motion state, which is known as multi-motion segmentation. The same motion label is assigned to features that belong to the same cluster. This process can be performed using subspace clustering methods or motion model-fitting methods.

Subspace clustering methods: The trajectories of the tracking feature points from a single rigid motion will all lie in a linear subspace with at most four dimensions when considering the affine camera model. Therefore, the multi-motion segmentation problem can be considered as a subspace clustering problem [78]. Assigning points to proper subspaces and estimating subspace parameters should be conducted simultaneously. Zhang et al. [79] proposed a clustering method for a permutation space. First, initial hypotheses were generated via random sampling. The permutation preferences of the points were then extracted and used for linkage clustering. New hypotheses were generated by randomly sampling each cluster. This sampling and clustering process was conducted iteratively until convergence was achieved. Based on this clustering method, Wang et al. [44] constructed a pipeline for dynamic SLAM that does not use semantic cues. Additionally, efficient dimension reduction can improve the performance of subspace clusters. For example, TMPCA [80] is an efficient data dimension reduction technique. Because it uses a smaller number of parameters than neural network (NN)-based models, it requires relatively few computations, which is important for ensuring real-time performance.

Motion model fitting methods: To some extent, a motion model fitting method is a special form of subspace clustering method. Unlike subspace clustering methods, motion model fitting methods directly estimate the motion matrix of feature correspondences. The types of motion models include the fundamental matrix, affine fundamental matrix, essential matrix, homography/projectivity, and affinity. The correspondences that fit the same motion model are grouped into clusters. Judd et al. [45] proposed a motion model fitting method for a 3D sensor (stereo camera, R-GBD camera, or lidar). They performed data association first and formed world- and image-space tracklet histories for each feature point. These tracklets could be segmented based on their observed motions, which is a multi-labeling problem.

#### Dynamic feature reconstruction and 3D object tracking

**Fig. 2:**
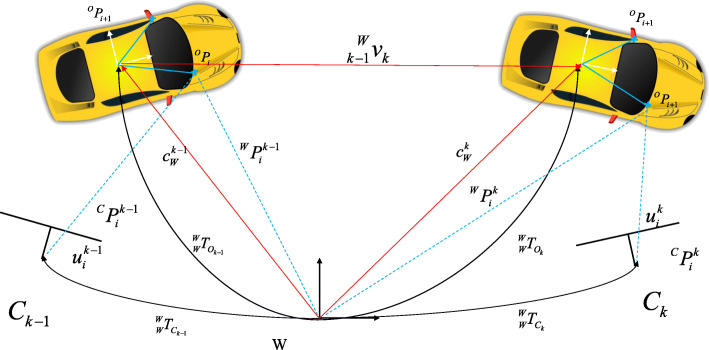
Model for a dynamic camera and dynamic object. The camera observes the same dynamic car at timestamps *k* − 1 and *k*. Here, the black solid curves represent camera ($_W^W{T}_c$) and object poses ($_W^W{T}_o$) in the world frame. Red solid lines represent the position and the speed of dynamic object in the world frame. Blue dashed lines represent 3D points in camera frames or the world frame

As shown in Fig. 2, the *i*-th point on a rigid object, denoted as ${}{}^O{P}_i$, has the same coordinates in the object coordinate system at different timestamps.

1 $$ ^O{P}_i =_W^W{T_{O_k}^{-1}}^W{P}_i^k =_W^W{T_{O_{k-1}}^{-1}}^W{P}_i^{k-1} $$

Here, $^W{P}_i^k={\left(X,Y,Z,1\right)}^{\mathrm{T}}\in {\mathbb{R}}^4$ denotes the homogeneous coordinate of the *i*-th (right subscript) 3D point (*X*, *Y*, *Z*)^T^ on a rigid object, as shown in the world frame (left superscript) at the *k*-th (right superscript) timestamp. The general notation $_W^W{T}_{O_k}\in SE(3)$ $$ \left(T:= \left[\begin{array}{c}R\kern1em t\\ {}0\kern1em 1\end{array}\right]\right) $$, where *R* ∈ *ℝ*^3 × 3^ denotes a rotation matrix and *t* ∈ *ℝ*^3^ denotes a translation vector, rather than $^W{T}_{O_k}\in SE(3)$, is used to denote the pose of an object with respect to the world frame at timestamp *k* in this survey. The former notation can also represent the motion from the world frame (left subscript) to the object frame (right subscript), as shown in the world frame (left superscript) at timestamp *k*, whose inverse is represented by $_{O_k}^{O_k}{T}_W\in SE(3)$. The left superscript indicates the frame from which the transformation occurs. Additional information regarding this notation is provided in ref. [81]. Let ${u}_i^k$ denote the features corresponding to $^W{P}_i^k$ in the image space, *π* denote the projection model, and $_W^W{T}_{C_k}$ denote the camera pose with respect to the world frame. Then, the re-projection error for the dynamic features of a rigid object can be obtained using Eq. (2).

2 $$ e={u}_i^k-\pi \left({}_W^W{T_{C_k}^{-1}}_W^W{T_{O_k}}^O{P}_i\right) $$

This formulation makes it possible to optimize the poses of the cameras ($_W^W{T}_{C_k}$) and moving objects ($_W^W{T}_{O_k}$) jointly, as well as the positions of their 3D points [48]. Another relationship that can be derived from Eq. (1) is

3 $$ ^W{P}_i^k =_W^W{T_{O_k}}_W^W{T_{O_{k-1}}^{-1}}^W{P}_i^{k-1} =_{O_{k-1}}^W{T_{O_k}}^W{P}_i^{k-1} $$

Here, $_{O_{k-1}}^W{T}_{O_k} =_W^W{T_{O_k}}_W^W{T}_{O_{k-1}}^{-1}\in SE(3)$ represents the pose change from *k*−1 to *k*, as shown in the world frame *W*, which represents the motion of an object with no consideration for its pose. Therefore, a new re-projection error can be obtained as follows:

4 $$ e={u}_i^k-\pi \left({}_W^W{T_{C_k}^{-1}}_{O_{k-1}}^W{T_{O_k}}^W{P}_i^{k-1}\right) $$

Additionally, the speed of a moving object can be represented as follows:

5 $$ _{k-1}^W{v}_k={\left({}^W{c}_k -^W{c}_{k-1}\right)}_{1:3}={\left({}_{o_{k-1}}^W{T_{O_k}}^W{c}_{k-1} -^W{c}_{k-1}\right)}_{1:3} $$

where ^*W*^*c*_*k*_ denotes the homogeneous coordinates of the object center expressed in the world coordinate system at timestamp *k*.

For a stereo camera or RGB-D camera, the depths of dynamic points can be obtained in the current frame. Therefore, the motion of a rigid object can be estimated easily. Unlike standard SLAM in a static scene, Bescos et al. [48] used Eq. (2) to establish a tightly coupled multi-object tracking and SLAM system. Zhang et al. [51] and Henein et al. [82] introduced the new factor representation in Eq. (4) into the factor graph of static SLAM. In this manner, they estimated the motion of a rigid object without using that object’s pose. Similarly, Wang et al. [44] took advantage of a stereo camera. Specifically, they used the coordinates of points expressed in the camera coordinate system directly, rather than those expressed in the world coordinate system. They estimated the motion of the camera with respect to static and moving objects. Then, object motion was obtained by multiplying the camera motion, inverse of the camera motion with respect to a moving object, and initial pose of the object center.

For a monocular camera, the reconstruction of moving objects is a nontrivial task. There are two main difficulties in reconstruction and tracking processing. First, standard triangulation is not suitable for dynamic features because back-projected rays do not intersect. Second, the estimated trajectory of a moving object is ambiguous and recovered as a one-parameter family of trajectories relative to the trajectory of the camera, which is known as the problem of relative scale ambiguity [83, 84].

**Fig. 3:**
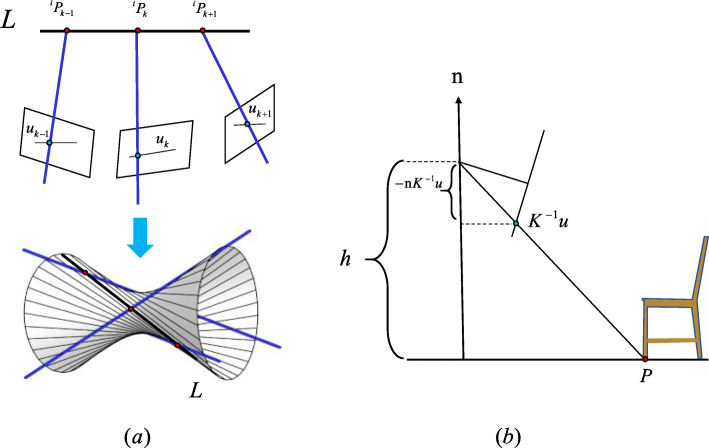
Approaches to addressing dynamic feature reconstruction for a monocular camera. (**a**): Trajectory triangulation with a line assumption. When the number of views *t* is three, the solution is a ruled surface. Therefore, to obtain a unique result, *t* must be at least five; (**b**): Illustration of reconstruction using the ground plane

The first difficulty can be overcome by incorporating additional motion constraints. Avidan and Shashua [85] assumed that point features move along an unknown 3D line, which is simply the original problem of finding a unique 3D line that intersects projected rays from *t* views (*t ≥ 5*), as shown in Fig. 3a. This method can work incrementally, but it requires several frames for each iteration. Although this method does not require any priors for camera motion, the specific form of object motion limits its application in the real world. For example, it cannot handle the features of a car winding along a flat road. Based on the fact that most objects move on flat planes, some methods [14, 46] reconstruct features *u* on the ground based on the current frame using the ground plane [**n**, h] (normal and distance in the camera frame), as shown in Fig. 3b.

6 $$ P=-\frac{h{K}^{-1}u}{{\mathbf{n}}^{\mathrm{T}}{K}^{-1}u} $$

Here, *K* denotes the camera intrinsic matrix and *P* denotes the 3D points corresponding to *p*. If the camera is fixed on a robot moving on the ground, the parameters of the ground plane can be obtained directly.

To tackle the second problem, Nair et al. [46] leveraged multiple sources to obtain localizations of moving objects and maintained cyclic consistencies in a pose graph. They first used the 3D coordinates of ground points obtained by Eq. (6) to estimate the camera ego motion scale and localizations of moving objects. Next, shape priors were used as another source of localization for moving objects. Finally, a pose graph was designed to maintain cyclic consistencies and solve the problem of relative scale ambiguity. Another approach to solving this problem was proposed by Qiu et al. [52] based on the fact that camera motion and object motion are independent. They quantified the correlation between camera motion and object motion, and formulated an objective function based on the quantification results to recover the scale factor of each tracking object.

Discussion: There are two main approaches to optimizing the trajectories of cameras and moving objects. One is called the separate or loosely coupled method, which optimizes the camera pose first and then optimizes dynamic object poses. The other is the joint or tightly coupled method, which optimizes the pose of the camera and dynamic objects simultaneously. Unlike the separate method, the joint method can maintain the motion consistency of moving objects and camera in a unified coordinate system.

Occlusion often occurs in SLAMMOT systems and it is more difficult to solve this problem compared to robust SLAM system (Robust SLAM section) because the estimated trajectories of the camera and moving objects may both drift or be lost due to occlusion caused by static objects or other moving objects. Additionally, when a lost object appears again, a new label is assigned to it if there is no special handling implemented, leading to a problem called label inconsistency. A general solution is to leverage historical information and establish associations between this information and current observations. Huang et al. [47] predicted cluster motion based on historical information during occlusion and associated it with re-detected observations. They then recovered the motion based on the information before and after occlusion.

## Using high-level features in dynamic SLAM

In contrast to low-level features, each high-level feature corresponds to a class of 3D objects. Compared to point features, high-level features are more discriminative and can handle low-texture scenes more easily [14]. It is worth noting that high-level features are not only used in object SLAM (using labeled objects as the elements of a map), but also in point-based SLAM. Representations of high-level features in the image space include the 2D bounding box representation and pixel-wise mask representation. The former can be extracted through object detection. The latter can be extracted using pixel-wise semantic segmentation. A detector for high-level features can be implemented using support vector machines (SVMs) [86], CRFs [87], and NNs [88]. Although mainstream detectors in the object detection field have recently been developed based on NNs, the SVM method is still worth considering for its lighter computations, which are important for achieving real-time dynamic SLAM performance.

For point-based SLAM, high-level features only serve as extra priors. Therefore, there is no need to complete data associations for high-level features. However, for object SLAM, data associations play an important role in object pose estimation. The essence of high-level data association is the MOT problem. Methods for MOT can be roughly divided into generative and discriminative methods. The current mainstream strategy is the discriminative method, which is also commonly referred to as tracking-by-detection or detection-based tracking. The main pipeline for this technique consists of four blocks for object detection, appearance modeling, metric learning, and data association. Regarding appearance modeling, various features are employed to describe objects such as features from accelerated segment testing (point features), optical flows (pixel patches), color histograms (region features), or learning-based features. Metric learning is closely related to appearance modeling. Its main task is to map features into new spaces and bring the features of the same object closer in space. The core of multi-object association is a maximum a posteriori problem that can be solved using CRFs, bipartite graph matching, or min-cost max-flow network flows. Additionally, one could predict bounding box positions using a filter-based method and match the results using the intersection over union.

Notably, the same low-level features, such as point features, can be used in both SLAM and MOT. This means that MOT can be embedded into the standard SLAM framework. By utilizing features in an appearance model, one can regroup high-level data associations into low-level-feature-based methods and learning-based methods.

Low-level-feature-based association methods: High-level associations can be established based on low-level features with the same labels. In terms of bounding box representations, additional information (such as trajectory and depth) is required to ensure that the correspondences between points and objects are valid because a bounding box always contains features that lie in the background and foreground. Yang and Scherer [14] constructed an object SLAM system utilizing ORB SLAM2. For static object features, they first associated point features with their corresponding high-level features (point-object associations). They then matched high-level features in different frames if they shared sufficient point features. Point-object associations should be constructed when points belong to an object. Therefore, simple bounding box constraints are inadequate. The authors added the constraints that points should be observed in a 2D bounding box for at least two frames and that they should be sufficiently close to the 3D box center. This method is different from the MOT pipeline described above because it leverages the camera pose to guide high-level associations implicitly. However, ambiguity exists in bounding box overlap areas. Additionally, descriptor-based methods perform well when an object is static or moving slowly, but it is difficult to track landmarks moving quickly in the image space. Therefore, Yang and Scherer [14] employed sparse optical flows to handle dynamic associations without using point positions. Huang et al. [47] elaborately established a probabilistic model to explore enhanced point-object associations for fast-moving objects. They proposed a heterogeneous CRF combining semantic, spatial, and motion information to associate features with landmarks and bounding boxes with clusters jointly, and then implemented the Kuhn-Munkres algorithm to match current clusters with previous clusters. For pixel-wise segmentation representations, a label must be assigned to each pixel in the mask. Wang et al. [44] completed this process at the superpixel level. Each superpixel is labeled with the label of the corresponding point feature. A K-nearest voting method was used for superpixels containing no labeled point features.

Learning-based methods: Li et al. [89] proposed a 3D object detection method for autonomous driving. They leveraged NNs directly to detect corresponding objects between pairs of stereo images. The key element of their method is the assignment of the union of left and right ground truth (GT) boxes (referred to as union GT boxes) as the target for object classification. Additionally, in their subsequent work [90], this concept was applied to perform data association between adjacent frames. This method can work well when an object moves slowly, which guarantees that there is a sufficient union region between the bounding boxes in adjacent frames. However, it cannot handle fast moving objects. Additionally, this method cannot handle occlusion well based on its simple matching procedure. Gordon et al. [53] designed a network that can handle temporary occlusion better based on the characteristics of long short-term memory [91].

### Robust SLAM

#### Using high-level features as semantic priors for low-level-feature-based SLAM

High-level features can guide motion segmentation for low-level features. Semantics can provide priors for representing the dynamic probabilities of features. However, it is ineffective to use semantic labels alone to define potential dynamic objects. For example, this method cannot classify books or chairs carried by an individual correctly because books and chairs are typically static from the perspective of semantics. Additionally, some background features may be contained inside bounding boxes. Therefore, additional information should be used to check each feature for robust motion segmentation.

For background points in bounding boxes, Ai et al. [34] utilized semantic information alone, but constructed a probability model for dynamic features and leveraged multiple observations to judge whether a feature was dynamic. They initialized the dynamic probabilities of ORB features based on semantic information and added the dynamic probability of a point if it was within a bounding box in a new observation. However, this method yields poor performance when dynamic objects move slowly because ground points may fall within bounding boxes for a long time. Zhang et al. [41] used a K-means clustering algorithm that considers depth information to distinguish foreground and background features in bounding boxes. Their method can work well under the assumption that the number of dynamic features in a box is greater than that outside the box. However, it is not suitable for complex scenes. Bescos et al. [26] used a CNN to perform pixel-wise segmentation on potential dynamic objects and then used geometric information to detect any dynamic features that were missed. All features labeled as potential dynamic objects were ignored in the ego motion estimation and map construction processes. This strategy can construct a more reliable map that can be reused in long-term applications. However, this makes a SLAM system more fragile when static features are culled based on semantic information (e.g., features on stationary cars). Ballester et al. [49] combined semantic and geometric information. They used geometric criteria to classify the potential dynamic objects detected by a CNN and applied static objects to ego motion and structure estimation. Compared to ref. [26], this method uses more static points to estimate local positions, but reduces the reliability of the map for long-term applications.

Discussion: Table 4 presents the performance improvements of some systems based on RGB-D cameras compared to ORB-SLAM2. All of the data in Table 4 were collected from the corresponding references. The absolute trajectory root-mean-squared error proposed in ref. [92] was used as a performance metric for comparison. These systems were tested on two types of sequences from the TUM RGB-D indoor dataset [92]. One type is a low-dynamic sequence called sitting (s), which contains only two sitting people. The other is a highly dynamic sequence called walking (w), which contains several walking people.

**Table 4:** Root-mean-squared error of ATE improvement for robust SLAM compared to ORB-SLAM2 on TUM datasets

	Low-level SLAM		Use high-level in point-based SLAM			
	Point-based		Point-based			Point-line-based
Year	2020	2020	2018	2018	2019	2019
References	Yang et al. [20]	Du et al. [22]	Besco et al. [26]	Yu et al. [28]	Cui and Ma [30]	Zhang et al. [41]
s_static	23.2%	–	–	25.9%	13.0%	24.1%
s_xyz	–	18.2%	−66.7%	–	–	3.1%
s_rpy	–	–	–	–	–	−15.8%
s_halfsphere	–	–	15.0%	–	–	58.6%
w_static	98.2%	94.9%	93.3%	97.9%	98.5%	98.3%
w_xyz	97.5%	95.6%	96.9%	96.7%	97.5%	97.7%
w_rpy	95.8%	93.8%	94.7%	48.7%	97.2%	76.4%
w_halfsphere	95.4%	92.7%	92.9%	93.76%	95.0%	96.7%

The results demonstrate that using high-level features in point-based SLAM can improve the accuracy of estimated trajectories. However, the improvement is not significant compared to some well-designed point-based SLAM methods [20]. Additionally, high-level feature extractors are time consuming and GPU dependent, which limits their application in computationally constrained cases. However, high-level features provide rich priors for systems that can be used to realize various important functions (e.g., background inpainting [26, 33], which is useful for AR applications, and maintaining long-term consistency without a probabilistic model [26]) in a relatively easy manner. Additionally, for low-level robust SLAM, historical observations must be utilized to maintain long-term consistency [22]. However, for SLAM systems using high-level features, long-term consistency can be guaranteed easily by culling all features that belong to some special semantics (e.g., pedestrians, cars, and riders) [26].

#### Using high-level features in object SLAM

For object SLAM, motion segmentation of high-level features can be completed using low-level features. An intuitive method is to determine the states of high-level features according to the number of static point features corresponding to them [14]. Additionally, optical flows can also be used to detect dynamic high-level features, whose processing is the same as that for low-level features. In contrast to low-level features, high-level features contain semantic information that can be used for motion segmentation.

After recognizing stationary high-level features in the image space, their corresponding 3D objects can be reconstructed and used to estimate camera ego motion. Existing 3D representations of objects in SLAM are grouped into parametric and nonparametric approaches. Parametric approaches represent an object using a regular 3D form such as cuboid [14] or dual quadric [93], whose parameters are tightly constrained by the 2D bounding box corresponding to the object. In contrast, nonparametric approaches reconstruct objects and represent them using a collection of small geometric structures such as surfels [55, 94, 95] or voluments [96]. Regarding limitations, parametric approaches ignore the details of objects, but incur lower computational costs. Nonparametric approaches describe objects in more detail, but require more memory and computations. Additionally, surfel representations are difficult to use directly for robotic tasks [96]. Cuboid representation is concretely discussed next.

A 3D box landmark can be represented by nine degrees of freedom (DoF) parameters (three DoF positions, three DoF rotations, and three DoF dimensions) and a semantic label. For a camera that can capture depth information in the current frame, the position and dimensions of a 3D box can be obtained from point cloud information. Gomez et al. [97] first calculated the maximum, minimum, and mean depth of objects. The depth of a vertex can be obtained from maximum and minimum values and the depth of a centroid can be obtained from mean values. However, this method cannot handle the case where the point that has the maximum depth is unobservable in the current frame. Wang et al. [44] leveraged the point clouds from many frames to recover the surface of an object first, and then estimated the position of its centroid. In terms of box orientation, it can be initialized to be vertical relative to the camera and tracked over time. For a monocular camera, Yang and Scherer [14] used vanishing points to sample many cuboid candidates. The best cuboid was then scored and selected based on image edges. Additionally, deep learning has been used to solve this problem. There are two main approaches to deep learning solutions.

First, one can generate a 3D point cloud and then detect objects based on this cloud [98]. Second, one can detect objects in the image space and then recover the 3D structures of those objects [89]. The former approach always requires two or more subnetworks and the latter approach relies heavily on 2D detection, which cannot make full use of 3D geometric information. Recently, Chen et al. [99] established an end-to-end method to estimate depth and detect 3D objects jointly. They encoded 3D geometry and semantic information by transforming a plane-sweep volume into a 3D geometric volume that bridges the gap between 2D images and 3D space.

Discussion: Unlike low-level features, high-level features can guide motion segmentation with priors. Additionally, using objects as elements in a map can provide long-range geometric and scale constraints for camera pose estimation [14]. Furthermore, a manageable and expressive map can be constructed using objects as elements. Using 3D boxes around objects as elements significantly reduces the number of parameters saved in a map, which is essential for large-scale applications. Gomez et al. [97] proposed a pose graph based on objects to update and manage a map for low-dynamic environments. An object landmark was parameterized as a nine-DoF 3D box with a semantic label and a probability that represents the object’s movability. When multiple mapping sessions are completed, the resulting maps are merged to form a new robust map. Unlike the map constructed by Bescos et al. [26] in Using high-level features as semantic priors for low-level-feature-based SLAM section, this map can be reused in long-term applications without losing any useful information.

### SLAMMOT

#### Using high-level features in point-based SLAM

**Fig. 4:**
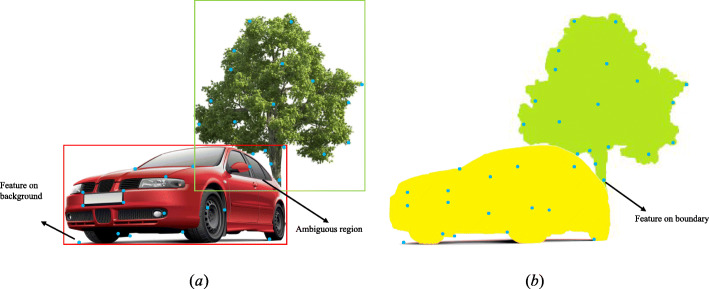
Two ways to perform multi-motion segmentation using semantic information: (**a**) Assigning semantic labels with bounding boxes and (**b**) assigning semantic labels with pixel-wise semantic masks

In contrast to applying clustering algorithms to low-level features, high-level features facilitate the clustering of map points belonging to independent objects with different dynamics, as well as the potential for detecting dynamic objects in one shot [100]. For rigid objects, features with the same semantic label always have the same motion label. As mentioned previously, semantic labels can be assigned to 2D bounding boxes or pixel-wise masks. A bounding box contains both object and background features. Additionally, there is ambiguity when features fall inside the union of two bounding boxes, as shown in Fig. 4a. Therefore, it is necessary to use geometric information (e.g., trajectory or depth information) to identify the features that actually lie on objects [47]. In contrast, methods based on semantic masks are easier to use because any feature that lies within a semantic mask belongs to the corresponding object. However, points falling on boundaries (Fig. 4b) may introduce errors in the trajectory and structure estimation process. Therefore, these features must be examined further or culled.

Discussion: Compared with the method using low-level features discussed in Multi-motion segmentation section, semantics-based methods are easier to implement and can be used in some high-level applications. However, they are not sufficiently robust for most practical environments because many objects have no labels in the real world [44]. Additionally, it is difficult to handle non-rigid objects when an object has more than one motion label. In contrast, subspace clustering methods and model fitting methods can cluster features without relying on semantic cues. Furthermore, they can handle non-rigid objects.

#### Using high-level features in object SLAM

For rigid objects, SLAMMOT based on high-level features does not need to perform multi-motion segmentation iteratively because each high-level feature corresponds to an object in the 3D world. Therefore, the core problem is how to establish high-level data associations and estimate the trajectories of objects in 3D space.

Yang and Scherer [14] leveraged vanishing points and ground planes to recover 3D boxes on the ground using a monocular camera. High-level data associations were established using a low-level-feature-based method. However, to avoid the relative scale problem, they only focused on estimating the relative poses of 3D landmarks in every frame, rather than estimating the trajectories of objects with respect to the world frame. Therefore, they could not use trajectory information to predict the poses of moving objects, which limited their ability to handle occlusion.

Qiu et al. [52] leveraged a NN to detect [54] and associate [53] high-level features. Next, 3D object motion was recovered from 2D object regions using region-based BA, which represents the relative motion between two dynamic frames (object frame and camera frame), as discussed in Dynamic feature reconstruction and 3D object tracking section. Finally, they solved the relative scale problem using independence analysis.

Existing data association and pose estimation approaches focusing on objects [14, 55, 101] are not sufficiently accurate or robust to handle complex environments containing multiple moving object instances. The combination of object SLAM and MOT is a novel and challenging research direction that requires further attention.

## Conclusions

For robust SLAM, high-level features (semantically labeled bounding boxes or pixel-wise masks) can provide low-level-feature-based SLAM with semantic priors to facilitate motion segmentation. Conversely, static high-level features can be matched and detected using low-level features. In terms of reconstruction and mapping, a parametric high-level landmark representation reduces the storage demands for maps. Additionally, semantic information makes a map more understandable. Regarding the accuracy of reconstruction and camera ego motion, SLAM based on high-level features alone is not as powerful as that based on low-level features in a static environment [93]. However, using these two levels of features together can result in better performance [47].

For SLAMMOT, dynamic data association is a very important task. However, standard descriptor-based methods cannot handle this task well because the guide matching technique (leveraging the poses of cameras and positions of 3D points to guide data association) [21] is invalid when dynamic objects move quickly. Most existing approaches leverage optical flows to address this problem. However, such flows are sensitive to illumination changes, which limit their application in real-world scenarios. Therefore, utilizing learning-based methods to extract more robust features and complete data associations is a promising alternative approach. Additionally, the proper probabilistic treatment of data associations is a valid method for robust tracking and mapping in dynamic scenes [47, 55]. Low-level-feature-based methods must perform a step of multi-motion segmentation to register features into clusters, which typically incurs a high computational cost. In contrast, object SLAM can skip this step by leveraging semantic information. The core of dynamic 3D object tracking is trajectory estimation. For 3D sensors, the poses and scales of objects or clusters can be easily obtained because depth features are available for each frame. However, for monocular cameras, additional work must be performed to overcome the problem of relative scale ambiguity.

Although SLAM based on high-level features and landmarks is more similar to human cognition, low-level features play an important role in accurate pose estimation. Therefore, for robot applications, using both types of features may be the best method.

## Data Availability

The datasets used and/or analyzed during the current study are available from the corresponding author on reasonable request.

## Abbreviations

SLAM: Simultaneous localization and mapping
MOT: Multiple object tracking
V-SLAM: Visual SLAM or vision-based SLAM
IMU: Inertial measurement unit
RGB-D: RGB-depth
BA: Bundle adjustment
3D: Three-dimensional
2D: Two-dimensional
SVM: Support vector machine
CRF: Conditional random field
NN: Neural network
DoF: Degree of freedom
GT: Ground truth
AR: Augmented reality
